# GABA-Induced Seizure-Like Events Caused by Multi-ionic Interactive Dynamics

**DOI:** 10.1523/ENEURO.0308-24.2024

**Published:** 2024-10-25

**Authors:** Zichao Liu, Erik De Schutter, Yinyun Li

**Affiliations:** ^1^School of Systems Science, Beijing Normal University, Beijing 100875, China; ^2^Computational Neuroscience Unit, Okinawa Institute of Science and Technology Graduate University, Okinawa 904-0495, Japan

**Keywords:** bifurcation, chloride, epilepsy, GABA stimuli, inhibition and excitation, ion dynamics

## Abstract

Experimental evidence showed that an increase in intracellular chloride concentration $\left({\left[Cl^{-}\right]}_{i}\right)$ caused by gamma-aminobutyric acid (GABA) input can promote epileptic firing activity, but the actual mechanisms remain elusive. Here in this theoretical work, we show that influx of chloride and concomitant bicarbonate ion $\left({\mathrm{HCO}}_{3}^{-}\right)$ efflux upon GABA receptor activation can induce epileptic firing activity by transition of GABA from inhibition to excitation. We analyzed the intrinsic property of neuron firing states as a function of $[Cl^{-}{]}_{i}$. We found that as $[Cl^{-}{]}_{i}$ increases, the system exhibits a saddle–node bifurcation, above which the neuron exhibits a spectrum of intensive firing, periodic bursting interrupted by depolarization block (DB) state, and eventually a stable DB through a Hopf bifurcation. We demonstrate that only GABA stimuli together with ${\mathrm{HCO}}_{3}^{-}$ efflux can switch GABA's effect to excitation which leads to a series of seizure-like events (SLEs). Exposure to a low $[K^{+}{]}_{\mathrm{bath}}\;$ can drive neurons with high concentrations of $[Cl^{-}{]}_{i}$ downward to lower levels of $[Cl^{-}{]}_{i}$, during which it could also trigger SLEs depending on the exchange rate with the bath. Our analysis and simulation results show how the competition between GABA stimuli-induced accumulation of $[Cl^{-}{]}_{i}$ and $[K^{+}{]}_{\mathrm{bath}}$ application-induced decrease of $[Cl^{-}{]}_{i}$ regulates the neuron firing activity, which helps to understand the fundamental ionic dynamics of SLE.

## Significance Statement

Epileptic seizures are known to be induced by disbalance between excitation and inhibition. However, how inhibition fails and why pyramidal neurons generate uncontrolled firing activities is not well known. We characterize how neuron firing activities are affected by dynamics of intracellular chloride $[Cl^{-}{]}_{i}$ and discover the conditions under which GABA stimuli could become excitatory instead of inhibitory. Neuron firing patterns crucially depend on the competition of two opposing effects: one is the GABA input-induced accumulation of $[Cl^{-}{]}_{i}$, and the other is the application of low potassium bath-induced decrease of $[Cl^{-}{]}_{i}$; this mechanism has not been addressed before. Our work helps to understand why and when inhibition fails in epileptic generation and how to prevent such epileptic episodes.

## Introduction

Epilepsy is characterized by recurrent, spontaneous brain seizures and is well known to be triggered by an imbalance between excitation and inhibition (Engel, 1996; McCormick and Contreras, 2001; Ziburkus et al., 2006; Ben-Ari et al., 2007; Jiruska et al., 2013; Grone and Baraban, 2015; Staley, 2015; Wenzel et al., 2023), but detailed mechanisms are still unclear. Each seizure episode is marked by abnormal neuronal discharge patterns (Buchin et al., 2018; Wenzel et al., 2019) and interneurons play an important yet controversial role in its induction (Yekhlef et al., 2015; Neumann et al., 2017). On the one hand, interneurons release gamma-aminobutyric acid (GABA) to excitatory neurons through GABAergic synapses, allowing influx of chloride and inhibiting the neuron's firing activity, thus reducing the likelihood of seizure (Trevelyan et al., 2007; Cammarota et al., 2013; Liou et al., 2018; Parrish et al., 2019). On the other hand, GABAergic stimulation can also trigger seizure generation (Perreault and Avoli, 1988; Staley et al., 1995; Kaila et al., 1997; Voipio and Kaila, 2000; Cohen et al., 2002; Gulledge and Stuart, 2003; Sipilä et al., 2005; Gnatkovsky et al., 2008; Fujiwara-Tsukamoto et al., 2010; Jedlicka et al., 2011; Sessolo et al., 2015; Librizzi et al., 2017; Elahian et al., 2018; Burman et al., 2019; Desroches et al., 2019; Rich et al., 2020; Lemaire et al., 2021; van Hugte et al., 2023; Weiss, 2023). However, the functional role of GABA in triggering SLE is complicated and not well understood. Indeed, substantial intracellular chloride accumulation has been observed in conjunction with epileptiform activity (Kaila and Voipio, 1987; Payne et al., 2003; Blaesse et al., 2009; Raimondo et al., 2012, 2015; Accardi, 2015; Doyon et al., 2016). Therefore, it is crucial to investigate the functional role of intracellular chloride concentration in regulating epileptic activity at a single neuron level comprehensively.

GABA receptor activation allows both $Cl^{-}$ influx and efflux of bicarbonate ion $\left({\mathrm{HCO}}_{3}^{-}\right)$ (Kaila, 1994; Kaila et al., 1997; Farrant and Kaila, 2007; Kim et al., 2009). ${\mathrm{HCO}}_{3}^{-}$ is produced by carbonic anhydrases using carbon dioxide (CO_2_) to regenerate intracellular ${\mathrm{HCO}}_{3}^{-}$ (Lambert and Grover, 1995; Voipio, 1998; Staley and Proctor, 1999). Its production is regulated by pH value (Kaila and Voipio, 1987; Voipio and Ballanyi, 1997), glial cells (Halassa and Haydon, 2010; Matos et al., 2018; Erhardt et al., 2020; Untiet et al., 2023), and the blood–brain barrier (Hladky and Barrand, 2016). The combined dynamics of chloride influx and ${\mathrm{HCO}}_{3}^{-}$ efflux upon GABA receptor activation, together with concurrent increase of extracellular potassium concentrations (McBain, 1994; Shin et al., 2010; de Curtis et al., 2018; González et al., 2019), in regulating seizure activity has not been fully explored.

While seizures have traditionally been recognized as network events (Traub and Wong, 1982; Fröhlich et al., 2006, 2010; Lytton, 2008; Liou et al., 2020; Otárula and Schuele, 2020), biophysical models have shown that contributing mechanisms are inherent to single neurons (Kager et al., 2000; Jiang et al., 2005; Ullah et al., 2009; Ullah and Schiff, 2010; Barreto and Cressman, 2011; Krishnan and Bazhenov, 2011; Volman et al., 2012; Wei et al., 2014; Călin et al., 2021; Lemaire et al., 2021; Currin and Raimondo, 2022). Previous theoretical studies assumed that factors such as ion concentrations (Chizhov et al., 2019; Lombardi et al., 2021; Gentiletti et al., 2022), oxygen levels (Bradbury and Davson, 1965), and dynamics of potassium (Traynelis and Dingledine, 1988; Durand et al., 2010; Contreras et al., 2021) exert influence over the voltage system, resulting in epileptic behaviors. Yet how the neuron firing activities are regulated by chloride dynamics and the relationship between chloride and potassium interplay in regulating epileptic activity have not been fully explored. Previous work (Krishnan and Bazhenov, 2011) included chloride dynamic without considering the contribution from ${\mathrm{HCO}}_{3}^{-}$ and focused more on the relationship between Na^+^ and K^+^ during seizure induction and termination, while less attention was paid to the relationship between the intracellular chloride dynamics and neural firing property or the interplay between chloride and potassium in regulating neuron seizure-like events (SLEs).

Our study focuses on the critical role of chloride dynamics in regulating epileptic activity and investigates the basic mechanisms of how a neuron's firing properties are shaped by the competition between opposing effects of chloride upon GABA stimuli and application of extracellular potassium bath.

## Materials and Methods

We analyze the neuron's intrinsic firing states and stability properties with respect to the intracellular chloride $[Cl^{-}{]}_{i}$ including saddle–node (SN) bifurcation and Hopf bifurcation (HB) by using the XPPAUT software (https://sites.pitt.edu/∼phase/bard/bardware/xpp/xpp.html). To focus on the pivotal role of $[Cl^{-}{]}_{i}$ in regulating SLE, we exclude additional factors such as dynamic changes of the oxygen level (Wei et al., 2014), volume (Kager et al., 2000; Østby et al., 2009), or interaction with glial cells (Volman et al., 2012; Mederos and Perea, 2019; Palabas et al., 2022). Finally, we simultaneously use GABA stimuli and $[K^{+}{]}_{\mathrm{bath}}$ application to show how these two processes interact with the intrinsic properties of the system.

We have carried our study in three steps: (1) study the intrinsic property of the isolated neuron system showing SN and HB bifurcations without considering the diffusion of extracellular potassium nor the GABA stimuli, but only focusing on the role of chloride in regulating neuronal firing activity; (2) apply GABA stimuli with a step current or spike trains with different frequencies with or without ${\mathrm{HCO}}_{3}^{-}$ efflux; and (3) incorporate the exchange of extracellular potassium with a fixed $[K^{+}{]}_{\mathrm{bath}}$, and illustrate the synergistic interplay between chloride and potassium in regulating different firing patterns.

### Membrane potential and ion concentration dynamics

We employ a single-compartment Hodgkin–Huxley-like model to simulate the neuron's action potentials (Hodgkin and Huxley, 1952; Traub et al., 1991; Wei et al., 2014). This model encompasses transient sodium currents, delayed rectifier potassium currents, as well as specific leak currents for sodium, potassium, and chloride ions along with Na^+^/K^+^ pump current. The dynamic equation governing the neuron's membrane potential V(t) later includes the stimuli from GABA, which can be expressed in Equation 1:

$$\begin{array}{rl} C\frac{dV}{dt}= & -g_{K}n^{4}(V-E_{k})-g_{\mathrm{Na}}m^{3}h(V-E_{\mathrm{Na}})-g_{\mathrm{KL}}(V-E_{K\;})-g_{\mathrm{NaL}}(V-E_{\mathrm{Na}})\\ & -g_{\mathrm{ClL}}(V-E_{\mathrm{Cl}})-\frac{\rho_{\mathrm{pump}}}{\gamma }+g_{\mathrm{GABA}}(V-E_{\mathrm{Cl}})+0.2\;*\;g_{\mathrm{GABA}}(V-E_{\mathrm{HC}O_{3}}), \end{array}$$

where, C is the membrane capacitance with a constant value; $g_{K}$ and $g_{\mathrm{Na}}$ represent the maximum conductance of voltage-gated potassium and sodium channels, respectively; $g_{\mathrm{KL}}$, $g_{\mathrm{NaL}}$, and $g_{\mathrm{ClL}}$ represent the conductance of leak potassium, leak sodium, and leak chloride channels.

The Na^+^/K^+^ pump actively transports two K^+^ ions into the cell and expels three Na^+^ ions out of the cell. Its impact on the membrane voltage is represented by Equation 2 (Wei et al., 2014):

$$\rho_{\mathrm{pump}}=\frac{\rho }{1.0+\exp\;\left(\frac{25-{\left[Na^{+}\right]}_{i}}{3}\right)}\times \frac{1.0}{1.0+\exp\;(3.5-{\left[K^{+}\right]}_{o})}\;,$$

where ρ represents the maximum strength of Na^+^/K^+^ pump. The constant $\gamma =S/(Fv_{i})$ serves to convert the dimension of current $\left(\mu A/cm^{2}\right)$ into the transport rate of ion concentration (mM/s), where S, $v_{i}$, and F represent the surface area of the cell, intracellular volume, and Faraday constant, respectively.

The last two terms in Equation 1 represent the contribution of GABA stimuli into the system, where GABA input can be a step current input:

$$g_{\mathrm{GABA}}=\mathrm{constant},\;t\in [t_{0},t_{n}],$$

or a spike train input with different frequencies:

$$g_{\mathrm{GABA}}=A_{0}\times \underset{i}{\sum }\;\left[\exp\;\left(-\frac{t-t_{i}}{\tau_{1}}\right)+\exp\;\left(-\frac{t-t_{i}}{\tau_{2}}\right)\right]\delta (t-t_{i}),$$

the reversal potential of GABA is calculated as follows:

$$E_{\mathrm{GABA}}=\frac{E_{\mathrm{Cl}}\;+0.2\;E_{\mathrm{HC}O_{3}}}{1+0.2}\;,$$

where we assume a constant reversal potential of ${\mathrm{HCO}}_{3}^{-}$, i.e., $E_{\mathrm{HC}O_{3}}=-13\;\mathrm{mV}\;$ (Payne et al., 2003). The concentration of ${\mathrm{HCO}}_{3}^{-}$ inside and outside of neuron has complex dynamics. When the GABA receptor is activated, ${\mathrm{HCO}}_{3}^{-}$ efflux will be counteracted by the absorption of CO_2_ inside the neuron, and the pH level can also modify ${\mathrm{HCO}}_{3}^{-}$ concentration (Kaila and Voipio, 1987; Kaila et al., 1997). The speed of absorbing CO_2_ is fast and the concentration of ${\mathrm{HCO}}_{3}^{-}$ was assumed to be constant in previous work (Kaila and Voipio, 1987; Staley et al., 1995; Staley and Proctor, 1999). To simplify the process, we assume that the conductance of GABA receptor for ${\mathrm{HCO}}_{3}^{-}$ is 20% of that to chloride ions, and the reversal potential of ${\mathrm{HCO}}_{3}^{-}$ is kept at an equilibrium state of −13 mV.

The activation and inactivation variables of voltage-gated sodium and potassium channels, denoted as *m*, *n*, and *h*, exhibit values ranging from 0 to 1. These variables represent the fraction of ion-selective channels in their closed and open states, as described in Equation 3:

$$\frac{dm}{dt}=\alpha_{m}(V)(1-m)-\beta_{m}(V)m;$$

$$\frac{dn}{dt}=\alpha_{n}(V)(1-n)-\beta_{n}(V)n;$$

$$\frac{dh}{dt}=\alpha_{h}(V)(1-h)-\beta_{h}(V)h.$$

In these equations, $\alpha_{i}\;,\;\beta_{i}$ denote the opening and closing rates governing the transitions of each ion channel state with i∈{m,n,h}, and these rates are dependent on the membrane potential V(t). The equations of $\alpha_{i}$ and $\beta_{i}$ in our model were derived from a model of hippocampal neurons (Traub et al., 1991; Gloveli et al., 2005). Further details are provided in Equation 4:

$$\alpha_{m}=\frac{0.32(V+54)}{1-\exp\;\left(-\frac{V+54}{4}\right)};\;\;\beta_{m}=\frac{0.28(V+27)}{\exp\;\left(\frac{V+27}{5}\right)-1};$$

$$\alpha_{h}=0.128\;\exp\left(-\frac{V+50}{18}\right);\;\beta_{h}=\frac{4}{1+\exp\;\left(-\frac{V+27}{5}\right)};$$

$$\alpha_{n}=0.032\frac{V+52}{1-\exp\;\left(-\frac{V+52}{5}\right)};\;\;\beta_{n}=0.5\exp\left(-\frac{V+57}{40}\right).$$

The reversal potentials for sodium, potassium, and chloride ions, denoted as $E_{\mathrm{Na}}$, $E_{K}$, and $E_{\mathrm{Cl}}$, are determined by the Nernst equation, as presented in Equation 5. Notably, these potentials evolve dynamically alongside changes in ion concentrations, distinguishing our approach from the Hodgkin–Huxley (HH) equations where ion concentrations are held constant, and the temperature is set as *T* = 310 K.

$$E_{Na}=26.64\;ln\left(\frac{{\left[Na^{+}\right]}_{o}}{{\left[Na^{+}\right]}_{i}}\right);\;E_{K}=26.64\;ln\left(\frac{{\left[K^{+}\right]}_{o}}{{\left[K^{+}\right]}_{i}}\right);$$

$$E_{Cl}=26.64\;ln\left(\frac{{\left[Cl^{-}\right]}_{i}}{{\left[Cl^{-}\right]}_{o}}\right).$$

The units, descriptions, and values of all parameters are comprehensively detailed in Table 1.

**Table 1.** The values of constant parameters used in the single-compartment model

Parameter	Description	Value
C	Membrane capacitance	$1\;\mu F\cdot cm^{-2}$
$g_{\mathrm{Na}}$	Maximal conductance of sodium current	$30\;\mathrm{mS}\cdot cm^{-2}$
$g_{K}$	Maximal conductance of potassium current	$20\;\mathrm{mS}\cdot cm^{-2}$
$g_{\mathrm{NaL}}$	Conductance of leak sodium current	$0.04\;\mathrm{mS}\cdot cm^{-2}$
$g_{\mathrm{KL}}$	Conductance of leak potassium current	$0.1\;\mathrm{mS}\cdot cm^{-2}$
$g_{\mathrm{ClL}}$	Conductance of leak chloride current	$0.1\;\mathrm{mS}\cdot cm^{-2}$
γ	Conversion factor	$0.03\;\mathrm{mM}\cdot cm^{2}\cdot s^{-1}\cdot \mu A^{-1}$
ρ	Maximal Na/K pump rate	$0.8\;\mathrm{mM}\cdot s^{-1}$
$U_{\mathrm{nkcc}1}$	Constant NKCC1 cotransporter strength	$0.1\;\mathrm{mM}\cdot s^{-1}$
$U_{\mathrm{kcc}2}$	Constant KCC2 cotransporter strength	$0.3\;\mathrm{mM}\cdot s^{-1}$
$\epsilon_{k}$	Maximal potassium diffusion rate	$0.25\;s^{-1}$
$[K^{+}{]}_{\mathrm{bath}}$	Normal bath potassium concentration	3.0mM
β	Ratio of the intra-/extracellular volume	7

### Ion concentration dynamics

The concentration of extracellular potassium $[K^{+}{]}_{o}$, intracellular sodium $[Na^{+}{]}_{i}$, and intracellular chloride $[Cl^{-}{]}_{i}$ dynamically change in response to the currents flowing through relevant ion channels and pumps, as elucidated in Equation 6:

$$\begin{array}{rl} \tau \frac{d{\left[K\right]}_{o}}{dt}= & \gamma \times (g_{K}n^{4}(V-E_{K})+g_{\mathrm{KL}}(V-E_{K}))-2\;*\;\rho_{\mathrm{pump}}-\rho_{\mathrm{NKCC}1}+\rho_{\mathrm{KCC}2}\\ & \;-\varepsilon_{K}({\left[K^{+}\right]}_{o}-{\left[K^{+}\right]}_{\mathrm{bath}}), \end{array}$$

$$\tau \frac{d{\left[\mathrm{Na}\right]}_{i}}{dt}=-\gamma \times (g_{\mathrm{Na}}m^{3}h(V-E_{\mathrm{Na}})+g_{\mathrm{NaL}}(V-E_{\mathrm{Na}}))-3\;*\;\rho_{\mathrm{pump}}+\rho_{\mathrm{NKCC}1},$$

$$\tau \frac{d{\left[\mathrm{Cl}\right]}_{i}}{dt}=\gamma \;*\;(g_{\mathrm{ClL}}(V-E_{\mathrm{Cl}})+g_{\mathrm{GABA}}(V-E_{\mathrm{Cl}}))+2\;*\;\rho_{\mathrm{NKCC}1}-\rho_{\mathrm{KCC}2}.$$

The extracellular potassium concentration $[K^{+}{]}_{o}$ changes by voltage-gated potassium channels, Na^+^/K^+^ pump, two cotransporters of NKCC1 (Na^+^/K^+^/2Cl^−^) and KCC2 (K^+^/Cl^−^) (Lauf and Adragna, 2000; Payne et al., 2003; Gamba, 2005; Kahle et al., 2008; Kaila et al., 2014; Moore et al., 2017), and the diffusion to the bath $[K^{+}{]}_{\mathrm{bath}}$, which is assumed to incorporate glia's function (Eq. 6a). $\varepsilon_{K}$ represents this diffusion rate. We initially exclude the extracellular potassium diffusion to comprehensively examine the dynamics of $[Cl^{-}{]}_{i}$ in regulating neuronal firing activity. The dynamics of intracellular sodium concentration $[Na^{+}{]}_{i}$ are regulated by the voltage-gated sodium current, the Na^+^/K^+^ pump, and NKCC1 cotransporter (Eq. 6b).

The dynamics of intracellular chloride concentration $[Cl^{-}{]}_{i}$ is regulated by the leak current, the Na^+^/K^+^/2Cl^−^ cotransporter NKCC1 (Eq. 7b) and the K^+^/Cl^−^ cotransporter KCC2 (Eq. 7a), and the influx current upon GABA receptor activation (Eq. 6c). The cotransporters NKCC1 and KCC2 are modeled in a Nernst equation like fashion (Østby et al., 2009; Wei et al., 2014):

$$\rho_{\mathrm{KCC}2}=U_{\mathrm{kcc}2}\ln\;\left(\frac{{\left[K^{+}\right]}_{i}{\left[Cl^{-}\right]}_{i}}{{\left[K^{+}\right]}_{o}{\left[Cl^{-}\right]}_{o}}\right),$$

$$\rho_{\mathrm{nkcc}1}=U_{\mathrm{nkcc}1}f({\left[K^{+}\right]}_{o})\left(\ln\;\left(\frac{{\left[K^{+}\right]}_{o}{\left[Cl^{-}\right]}_{o}}{{\left[K^{+}\right]}_{i}{\left[Cl^{-}\right]}_{i}}\right)+\ln\;\left(\frac{{\left[Na^{+}\right]}_{o}{\left[Cl^{-}\right]}_{o}}{{\left[{\mathrm{Na}}^{+}\right]}_{i}{\left[Cl^{-}\right]}_{i}}\right)\right),$$

$$f({\left[K^{+}\right]}_{o})=\frac{1}{1+\exp(16-{\left[K^{+}\right]}_{o})},$$

where $U_{\mathrm{kcc}2},\;U_{\mathrm{nkcc}1}$ are the constant strength of cotransporters.

By the electroneutrality condition (Wei et al., 2014; Raimondo et al., 2015), the intracellular potassium concentration $[K^{+}{]}_{i}$ and extracellular concentrations of sodium $[Na^{+}{]}_{o}$ and chloride $[Cl^{-}{]}_{o}$ can be calculated as follows:

$$[K^{+}{]}_{i}=100-[Na^{+}{]}_{i}+[Cl^{-}{]}_{i},$$

$$[Na^{+}{]}_{o}=135-\beta ({\left[Na^{+}\right]}_{i}-20),$$

$$[Cl^{-}{]}_{o}=145-\beta ({\left[Cl^{-}\right]}_{i}-6).$$

### Phase space, dynamic trajectory, and stationary analysis by nonlinear dynamics theory

To explore the stability properties of the coupled differential equations more comprehensively across the entire seven-dimensional space $\left(V,\;m,\;n,\;h,{\left[K^{+}\right]}_{o},{\left[Na^{+}\right]}_{i},\;{\left[Cl^{-}\right]}_{i}\right)$, we employed XPPAUT (Ermentrout, 2002) to calculate fixed-point solutions. To determine whether the steady-state solution is stable, we calculate the Jacobian's matrix's eigenvalues at each fixed-point solution, and the real part of the eigenvalue will be used to measure the stability of each fixed-point solution, negative values indicate stable solutions, and positive value indicate unstable solutions.

### Numerical methods

We employed the fourth-order Runge–Kutta method within MATLAB (MathWorks) to perform the numerical integration of the coupled differential equations. Access to both MATLAB and XPPAUT codes can be found on the GitHub platform: https://github.com/ICANPB/Chloride-and-epilepsy.

## Results

We present our results in three parts, investigating models of increasing complexity. In the first part, we illustrate how neuronal intrinsic firing activities are modulated by different levels of intracellular chloride $[Cl^{-}{]}_{i}$.

### Bifurcation analysis of neuronal firing activity properties dependence on $[Cl^{-}{]}_{i}$

In contrast to potassium, which has been extensively studied (Bazhenov et al., 2004; Cressman et al., 2009; Wei et al., 2014; Depannemaecker et al., 2022), the relationship between chloride dynamics and epileptic seizures, particularly the accumulation of $[Cl^{-}{]}_{i}$ during GABA stimulation of a neuron, has not received much attention in computational models. We first demonstrate how the dynamic property of neuron firing activity is regulated by intracellular chloride $[Cl^{-}{]}_{i}$, before considering external GABA input.

**Figure 1.**
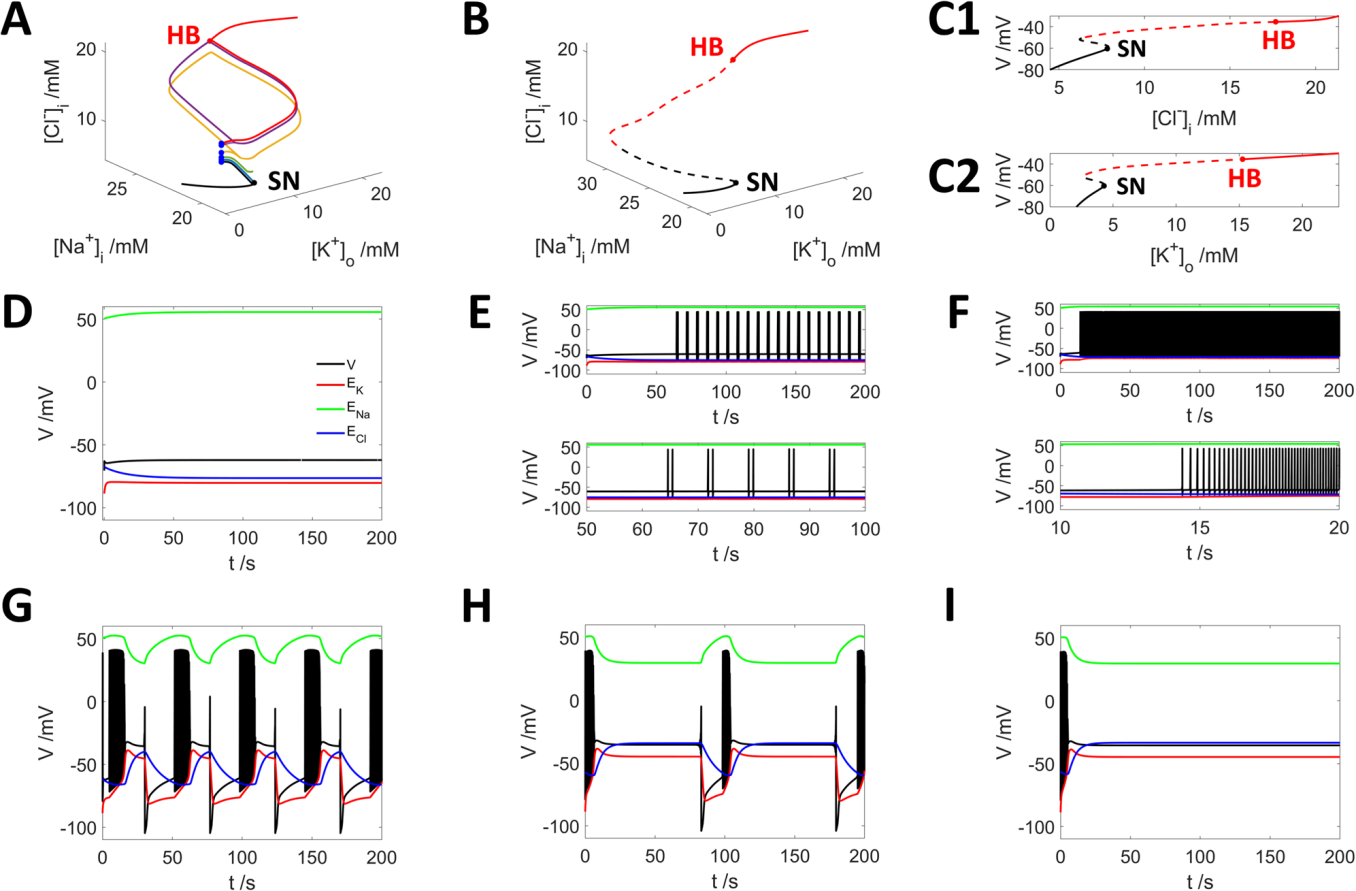
Different initial $[Cl^{-}{]}_{i}$ leads to varying firing activities with saddle–node (SN) bifurcation and Hopf bifurcation (HB) which characterizes the neuronal firing dynamics. ***A***, Dynamic traces in the phase space with different initial conditions of $[Cl^{-}{]}_{i}$. Solid black curve, dynamic traces to resting states; solid red curve, dynamic trace to depolarization block (DB); the blue dots, different initial conditions of $[Cl^{-}{]}_{i}$. ***B***, SN and HB bifurcations and fixed-point solutions of our model. Solid black curve below the saddle–node (SN) point: the stable resting state; solid red curve located above the HB point: stable DB states. Black dashed curve, fixed points with real eigenvalues; red dashed curve, fixed points with imaginary eigenvalues. ***C***, Fixed-point solutions projected onto the subspace of $[K^{+}{]}_{o}$ and voltage (***C1***) and onto the subspace of $[Cl^{-}{]}_{i}$ and voltage (***C2***). ***D–I***, Six examples of neuronal firing behaviors with increasing $[Cl^{-}{]}_{i}$ as initial conditions (with $[Na^{+}{]}_{i}=20.06\;\mathrm{mM}$, $[K^{+}{]}_{o}=2.99\;\mathrm{mM}\;$ unchanged). Within these panels, red, green, and blue curves correspond to the reversal potentials of potassium, sodium, and chloride, respectively. ***D***, Initial value of $[Cl^{-}{]}_{i}$ is smaller than 9.87 mM, the system relaxes to a resting state with $[Cl^{-}{]}_{i}=\;7.85\;\mathrm{mM}$. ***E***, Initial value of $[Cl^{-}{]}_{i}$ is set to 9.88 mM; it leads neuron to periodic bursting. ***F***, At an initial level of $[Cl^{-}{]}_{i}$ at 10.23 mM, neuron shows intensive firing activities. ***G***, An initial elevation of $[Cl^{-}{]}_{i}$ to 10.93 mM leads to periodic bursting with DB. ***H***, With an initial elevation of $[Cl^{-}{]}_{i}$ to 11.99 mM, the period of periodic bursting with DB is elongated. ***I***, Neuron evolves to DB state with an extremely higher value of $[Cl^{-}{]}_{i}$ of >12.27 mM. ***E–I*** share the same legend as ***D***.

Neurons evolve to distinct discharge behaviors with different initial concentrations of $[Cl^{-}{]}_{i}$ (Fig. 1*A*, blue dots). For instance, a neuron starting with a low value of $[Cl^{-}{]}_{i}=5.97\;\;\mathrm{mM}$ from resting state of −70.74 mV is stable at this resting state (Fig. 1*A*, black curve; Fig. 1*D*), while a neuron with much higher level of $[Cl^{-}{]}_{i}$ at 12.27 mM eventually reached a state of depolarization block (DB; Fig. 1*A*, red curve; Fig. 1*I*). In between, neurons with increasing elevation of $[Cl^{-}{]}_{i}$ exhibit periodic bursting (Fig. 1*E*), intensive spiking (Fig. 1*F*), periodic bursting interrupted with DB (Fig. 1*G*), and the duration of DB increases with higher $[Cl^{-}{]}_{i}\;$ (Fig. 1*H*).

To better understand how intracellular chloride concentration of $[Cl^{-}{]}_{i}$ shapes neuronal firing patterns, we found fixed-point solutions of the coupled differential equations in high-dimensional space by the XPPAUT software (Ermentrout, 2002) and analyzed their stability by calculating the eigenvalues of the Jacobian's matrix at each fixed-point solution. The distribution of fixed points in our dynamical system in the subspace of ion concentrations is depicted in Figure 1*B*. Our results show that an SN and an HB occur at $[Cl^{-}{]}_{i}=7.89\;\mathrm{mM}\;$ and $[Cl^{-}{]}_{i}=17.66\;\mathrm{mM}$, respectively. Furthermore, the SN bifurcation projected into the subspace of $[Cl^{-}{]}_{i}$ and membrane potential is shown in Figure 1*C2*. Neurons initiating with values lower than SN point of 7.89 mM evolve toward a resting state, whereas neurons with $[Cl^{-}{]}_{i}$ higher than the HB point of 17.66 mM are attracted to stable DB states. Neurons with values between the SN and HB points exhibit a variety of dynamic behaviors including tonic spiking and periodic bursting, corresponding to a set of limit cycles (Fig. 1*A*, light blue curves) in the phase space; referred to as “loop” structures (Barreto and Cressman, 2011). These “loop” structures show strong cyclic variation of $[Cl^{-}{]}_{i}$ and of $E_{\mathrm{Cl}}$ (Fig. 1*F*,*G*). The SN bifurcation was also observed in the subspace of potassium and membrane potential (Fig. 1*C1*), consistent with previous findings (Bazhenov et al., 2004; Cressman et al., 2009; Wei et al., 2014; Depannemaecker et al., 2022).

Therefore, neuron firing activity is attracted to different states, depending on the initial states of $[Cl^{-}{]}_{i}$. Next, we will explore how neuron firing activity is modified by GABA stimuli through the dynamics of $[Cl^{-}{]}_{i}$ and specify when GABA stimuli can switch its effect from inhibition to excitation and trigger SLEs in the neuron.

### Pure GABAergic stimuli inhibit neuron firing activity

**Figure 2.**
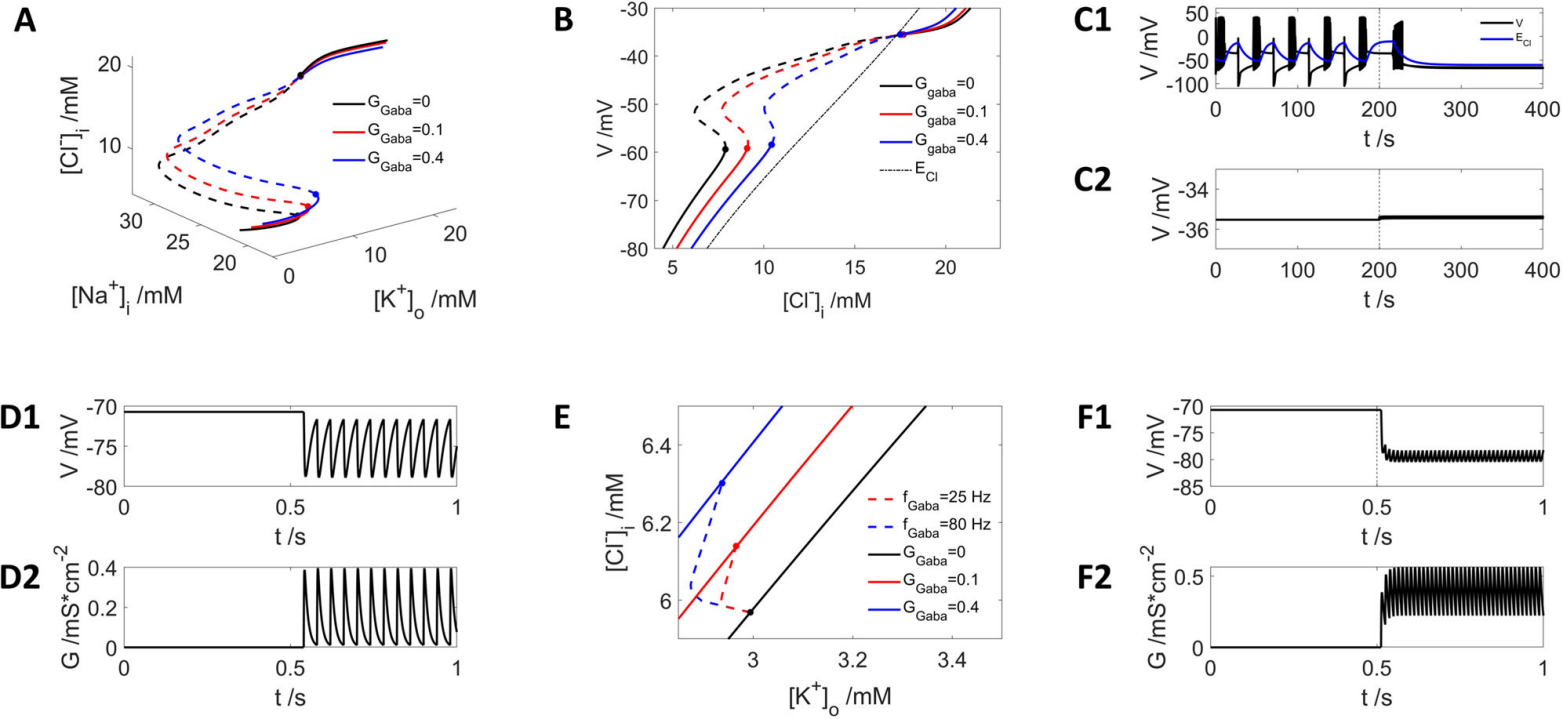
Exclusive GABA input leads to inhibition. ***A***, Distribution of the fixed-point solution of the neuron state with different step GABA current strengths: black, $G_{\mathrm{GABA}}=0\;\mathrm{mS}\cdot cm^{-2}$; red, $G_{\mathrm{GABA}}=0.1\;\;\mathrm{mS}\cdot cm^{-2}$; blue, $G_{\mathrm{GABA}}=0.4\;\mathrm{mS}\cdot cm^{-2}$. ***B***, Projection of the fixed-point solutions with different GABA current (as shown in ***A***) to the axis of $V-[Cl^{-}{]}_{i}$. Dotted black curve, reversal potential of chloride $E_{\mathrm{Cl}}$. ***C1***, A periodic bursting with DB neuron is inhibited by GABA inhibition at 80 Hz starting at *t* = 200 s (dashed vertical line), and the neuron evolves to its resting state. Blue curve: $E_{\mathrm{Cl}}$. ***C2***, An example of depolarizing effect of GABA with higher $[Cl^{-}{]}_{i}$ than the cross point shown in Figure 2*B*. ***D1***, A resting neuron is hyperpolarized by GABA inhibition at 25 Hz starting at *t* = 0.5 s. ***D2***, GABA spike train dynamics with 25 Hz. ***F1***, ***F2*** are similar to ***D1***, ***D2*** but with a frequency of 80 Hz. ***E***. Dynamic trajectory in the phase space for ***D1*** (red dashed curve) and ***F1*** (blue dashed curve).

We applied two patterns of GABA stimuli to the neuron: a step GABA current (Fig. 2*A*,*B*) or spike trains of GABA input with different frequencies (Fig. 2*C–F*).

With step GABA current input the neuron is mostly inhibited (Fig. 2*A*,*B*). As can be seen in Figure 2*A*, the corresponding $[Cl^{-}{]}_{i}\;$ at the SN bifurcation point increases with higher tonic GABA input (Fig. 2*A*, blue and red curves compared with the black curves), indicating the hyperpolarization effect of GABA. We computed under which condition the tonic GABA effect can switch from inhibitory to excitatory. By calculating the distribution of the stationary states of the neuron with GABA current input, the voltage can be plotted as a function of $[Cl^{-}{]}_{i}$ and compared with the reversal potential of chloride $E_{\mathrm{Cl}}$ for each state (Fig. 2*B*). The cross section between the two types of curves is near the HB point. This implies that only at very depolarized states the tonic GABA effect becomes depolarizing (Fig. 2*C2*), for all voltages below −35.72 mV GABA is inhibitory (Fig. 2*B*).

To demonstrate this principle, we applied GABA spike trains (Fig. 2*C–F*) to the neuron system. Continuous GABA stimuli of 25 Hz (Fig. 2*D*) and 80 Hz (Fig. 2*F*) hyperpolarize the membrane potential. Even starting from a higher initial $[Cl^{-}{]}_{i}$ corresponding to periodic bursting with DB state (Fig. 1*G*), stimulation by 80 Hz GABA input still causes inhibition (Fig. 2*C1*) and returns the neuron to a resting state. Even though $[Cl^{-}{]}_{i}\;$ was already very high, it is still inhibited by GABA and returns to a low level of $[Cl^{-}{]}_{i}\;$ corresponding to the resting state. The excitatory effect of GABA needs to satisfy the condition of $E_{\mathrm{Cl}}>V$, for example, if the neuron starts with a DB state (Fig. 1*I*), application of 80 Hz GABA stimuli causes a DB state at an even higher voltage (Fig. 2*C2*).

Our results demonstrate that GABA stimuli can lead to accumulation of $[Cl^{-}{]}_{i}\;$ (Fig. 2*E*); however, this does not lead to an excitatory effect because even though the reversal potential of chloride $E_{\mathrm{Cl}}$ increases by GABA-induced $[Cl^{-}{]}_{i}\;\;$ accumulation, it usually remains lower than steady state voltage values. In the next section we add the dynamics of ${\mathrm{HCO}}_{3}^{-}$ efflux upon GABA stimuli to investigate whether it will change the response to GABA activation.

### GABA stimuli with ${HCO}_{3}^{-}$ outflux trigger a spectrum of neural firing patterns evolving toward DB

**Figure 3.**
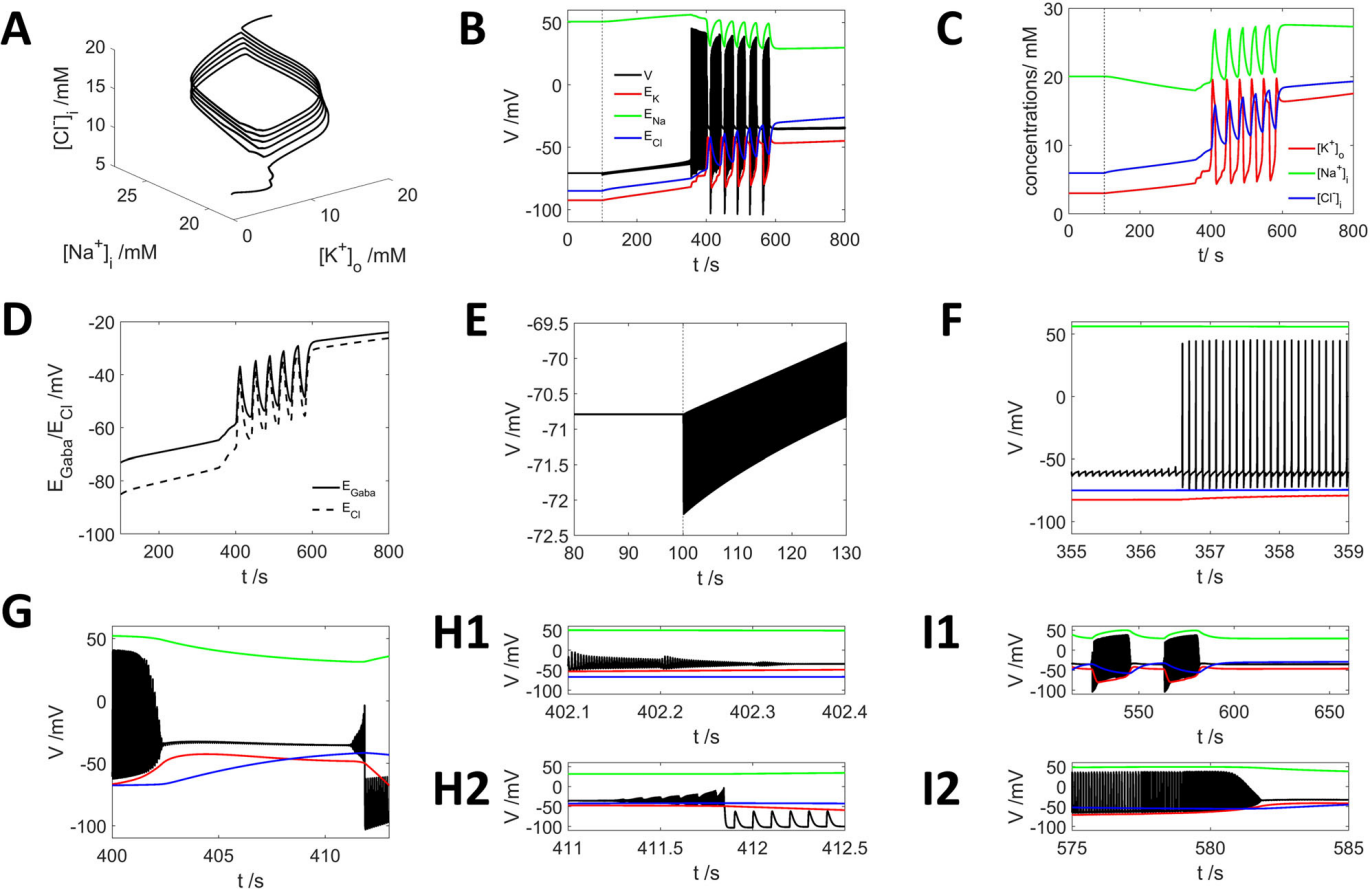
Neuron's intensive firing activity triggered by GABA spike stimuli with ${\mathrm{HCO}}_{3}^{-}$ outflux. ***A***, Dynamic trajectory of the neural firing activity in the phase space by 10 Hz GABA spike input and ${\mathrm{HCO}}_{3}^{-}$ outflux shows spinning up toward the higher $[Cl^{-}{]}_{i}$ direction. ***B***, Dynamics of neuron membrane potential (black curve) by 10 Hz GABA stimuli starting at *t* = 100 s (vertical dashed line) and reversal potential for each ion ( $E_{\mathrm{Na}}$, green; $E_{K}$, red; $E_{\mathrm{Cl}}$, blue), corresponding to dynamic trajectory shown in ***A***. ***C***, Dynamics of the ion concentrations. ***D***, Reversal potentials of chloride $E_{\mathrm{Cl}}\;$and GABA $E_{\mathrm{GABA}}$. ***F–I***, The voltage dynamics from ***B*** at different time points, showing the details of the voltage dynamics. ***F–I*** share the same legends as in ***B***.

Remarkably, 10 Hz GABA spike train stimulation combined with ${\mathrm{HCO}}_{3}^{-}$ outflux make the neuron generate a series of intensive firing activities evolving to periodic bursting with DB (Fig. 3*A*,*B*), which can be seen as a form of SLE. The dynamic trajectory of the neuron firing activity within the phase space evolves from the SN point to the HB point, encompassing all the intermediate states of limit cycles (Fig. 3*A*), exhibiting tonic firing (Fig. 3*G*), periodic bursting with DB (Fig. 3*H*), and it eventually evolves to a DB state (Fig. 3*I*).

For the initial period of GABA input, the GABA effect on the membrane potential is hyperpolarizing (Fig. 3*E*). At approximately *t* = 130 s, the GABA's effect turns to depolarizing but does not trigger action potentials (Fig. 3*B*); at approximately *t* = 356.7 s, the neuron begins to generate action potentials (Fig. 3*F*), indicating the GABA effect turns to excitatory. At approximately *t* = 360 s, neuron starts to exhibit pseudoperiodic bursting activities interrupted by DB (Fig. 3*A*,*B*,*G*) and with continuing input of GABA and ${\mathrm{HCO}}_{3}^{-}$ outflux, neuron finally evolves to a stable DB state at approximately *t* = 590 s (Fig. 3*I*). With the ${\mathrm{HCO}}_{3}^{-}$ outflux upon GABA receptor activation, the system reversal potential of GABA $E_{\mathrm{GABA}}$ is systematically higher than the reversal potential of chloride $E_{\mathrm{Cl}}\;$ (Fig. 3*D*).

Our simulation results demonstrate that evolution to SLE can result from $\mathrm{HC}O_{3}^{-}$ outflux upon GABA receptor activation. The basic mechanism is the high reversal potential of ${\mathrm{HCO}}_{3}^{-}$ $\left(E_{\mathrm{HC}O_{3}}=-13\;\;\mathrm{mV}\right)$ driving the neuron's membrane potential and pushing it toward higher $[Cl^{-}{]}_{i}$. These findings suggest a potential way in which GABA input can trigger epileptic seizures.

However, in mature animals, only in disease states GABA receptor activation will induce epileptic firing activities. This indicates that most of the time, ${\mathrm{HCO}}_{3}^{-}$'s effect is compensated by other mechanisms, one of which may be extracellular potassium homeostasis. Experimental evidence showed that when the extracellular potassium concentration is kept at a low level $\left({\left[K^{+}\right]}_{\mathrm{bath}}=3\;\mathrm{mM}\right)$, neurons stay at resting state (Kaila et al., 1997; Kofuji and Newman, 2004; de Curtis et al., 2018; González et al., 2018). In the next sections, we investigate the effect of applying an external potassium bath with $[K^{+}{]}_{\mathrm{bath}}=3\;\mathrm{mM}$ on neuron firing activity with simultaneous GABA stimuli with outflux of ${\mathrm{HCO}}_{3}^{-}$.

### Synergistic interplay between intracellular chloride and extracellular potassium results in different firing activities

Previous theoretical work (Wei et al., 2014) suggested that $[K^{+}{]}_{\mathrm{bath}}$ determines the neuronal steady state. We next investigate how $[K^{+}{]}_{\mathrm{bath}}$ affects the dynamic changes in $[Cl^{-}{]}_{i}$ during GABA stimuli and the resulting neuronal firing activity.

**Figure 4.**
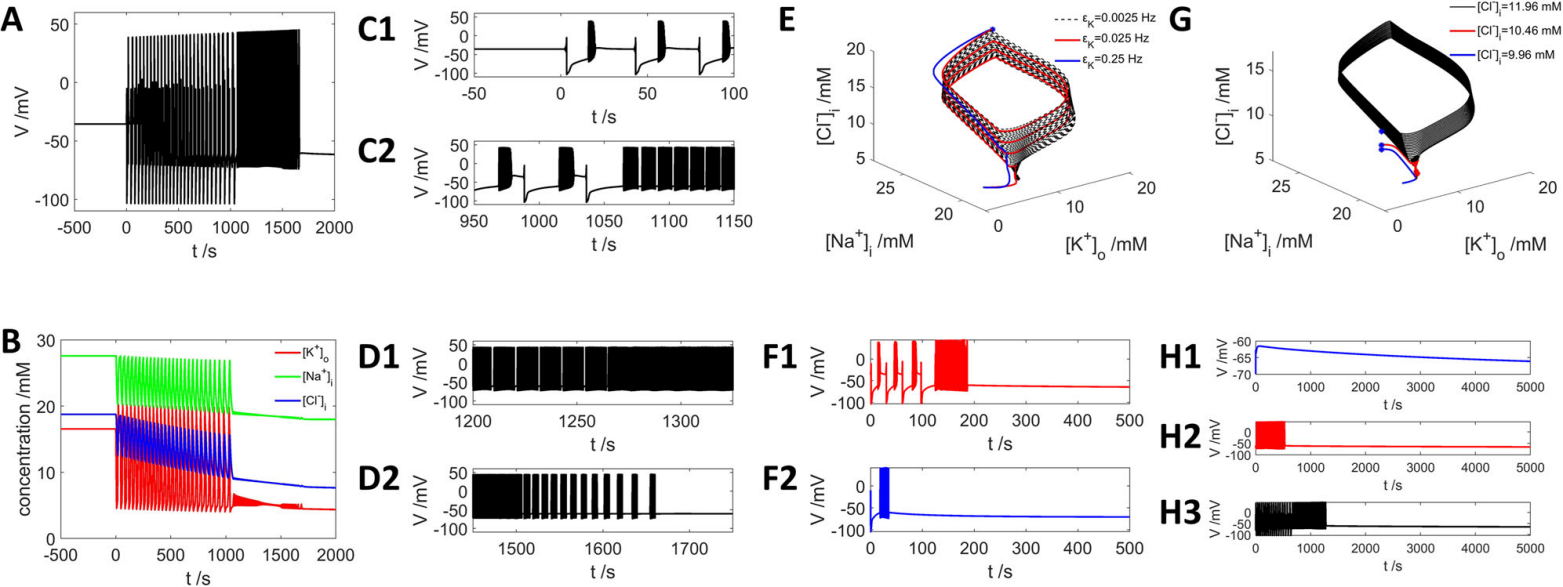
Extracellular potassium exchange with a low $[K^{+}{]}_{\mathrm{bath}}=3\;\mathrm{mM}$ drives the system to a resting state with low $[Cl^{-}{]}_{i}$. ***A***, Voltage dynamics from a DB state by adding $[K^{+}{]}_{\mathrm{bath}}=3\;\mathrm{mM}$ at *t* = 0 s. ***B***, Dynamics of ion concentrations in the process of ***A***. ***C1***, ***C2***, ***D1***, ***D2*** show more detailed plots of the typical firing patterns and their transitions in ***A***. ***E***, Dynamic trajectories in the phase space showing the voltage evolution from DB state to resting state for different exchange rates: black dashed curve with $\varepsilon_{k}=0.0025\;s^{-1}\;$(***A***), red curve with $\varepsilon_{k}=0.025\;s^{-1}$ (***F*1**), blue curve with $\varepsilon_{k}=0.25\;s^{-1}$ (***F*2**). ***G***, Dynamic trajectories showing that neuron with initial high level of $[Cl^{-}{]}_{i}$ (black curve, ***H*3**), middle level (red curve, ***H*2**), and low level (blue curve, ***H*1**) of initial $[Cl^{-}{]}_{i}$ are all driven to the resting state by $[K^{+}{]}_{\mathrm{bath}}=3\;\mathrm{mM}$.

First, we show that connecting a neuron in DB state with a high $[Cl^{-}{]}_{i}$ of 18.75 mM to a potassium bath with $[K^{+}{]}_{\mathrm{bath}}=3.0\;\mathrm{mM}$ successfully drives the neuron toward its resting state. During this transition process, the neuron exhibits a seizure-like event (Fig. 4*A*), meanwhile the dynamic trajectory spins down to a low $[Cl^{-}{]}_{i}$ (Fig. 4*E*, black dashed curve). Second, modulating the rate of K^+^ exchange with the bath (diffusion rate $\varepsilon_{k}$) strongly affects the neuron firing activity during its journey to the resting state (Fig. 4*E*,*F*). A slower exchange rate with $\varepsilon_{k}=0.025\;s^{-1}$ will cause long-lasting SLE before returning to the resting state (Fig. 4*E*,*F1*, red curves), while the neuron goes to resting state much faster with $\varepsilon_{k}=0.25\;s^{-1}$ (Fig. 4*E*,*F2*, blue curve), without experiencing periodic bursting with DB state. Third, we show that neurons starting with different initial levels of $[Cl^{-}{]}_{i}$ all evolve to the same resting state with $[K^{+}{]}_{\mathrm{bath}}=3.0\;\mathrm{mM}$ but exhibit different firing activities (Fig. 4*G*,*H*). Our findings suggest that $[Cl^{-}{]}_{i}$ and $[K^{+}{]}_{o}$ interact synergistically in regulating neuron firing activities, shedding light on the basic mechanism of interplay between potassium and chloride dynamics in regulating SLEs.

**Figure 5.**
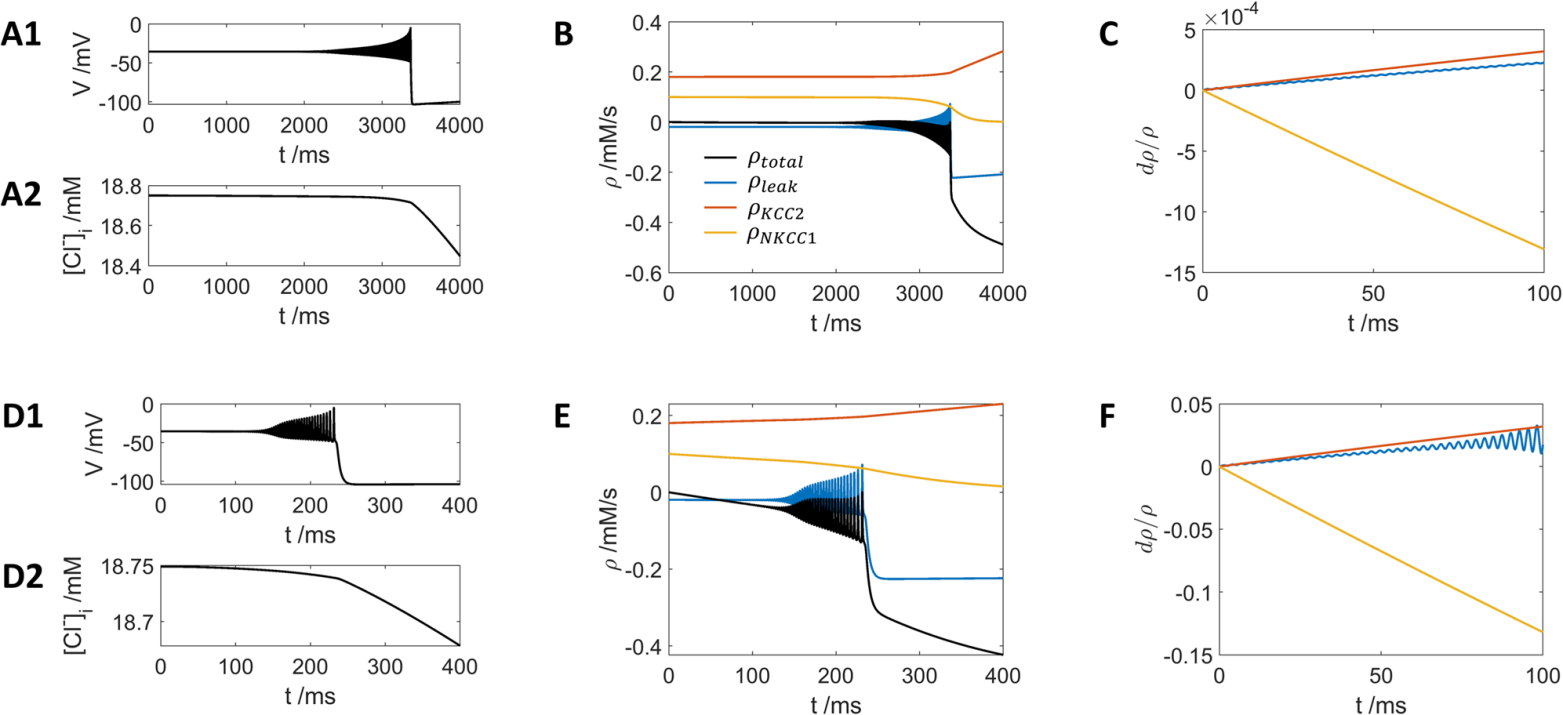
Detailed dynamic process showing how chloride concentration decreases during potassium bath application with $[K^{+}{]}_{\mathrm{bath}}=3\;\mathrm{mM}$. ***A–C*** show detailed dynamics from 0 to 4 s in Figure 4*C1*; ***D–F*** show detailed dynamics in Figure 4*F1* from 0 to 0.4 s. The red curves in ***B*** and ***E*** represent the contribution from KCC2, while the yellow curves in ***B*** and ***E*** represent the contribution from NKCC1 and the blue curves in ***B*** and ***E*** represent the leak current of chloride. The black curves in ***B*** and ***E*** represent the summation of these three currents. ***C***, ***F***, Change of the same three currents relative compared with the original values at *t* = 0.

As can be seen in Equation 6c, the concentration variation of intracellular chloride depends on the cotransporters’ contribution (Eqs. 7a, 7b). As $[K^{+}{]}_{o}$ decreases, the contribution of $\rho_{\mathrm{nkcc}1}$ decreases, and that of $\rho_{\mathrm{KCC}2}$ increases, both of which decrease $[Cl^{-}{]}_{i}$. Meanwhile, when $[K^{+}{]}_{o}$ decreases, the term of $\gamma \;*\;(g_{\mathrm{ClL}}(V-E_{\mathrm{Cl}}))$ also tends to decrease; therefore, all three factors contribute to decreasing $[Cl^{-}{]}_{i}$. Figure 5, *A–C*, shows the detailed contribution of cotransporters and leak chloride current to chloride decrease in Figure 4*C1*, where $\varepsilon_{k}\;=0.0025\;s^{-1}$. The increase of KCC2 current (Fig. 5*B*, red curve) and decrease of NKCC1 (Fig. 5*B*, yellow curve) current both diminish $[Cl^{-}{]}_{i}$; meanwhile, the leak current (Fig. 5*B*, blue curve) also decreases. Similarly, Figure 5, *D–F*, shows the details of how the $[K^{+}{]}_{o}$ drives the $[Cl^{-}{]}_{i}\;$ decrease with much larger $\varepsilon_{k}=0.25\;\;s^{-1}$, corresponding to Figure 4*F1*.

Our initial investigation of the effect of $[K^{+}{]}_{\mathrm{bath}}=3.0\;\mathrm{mM}$ had no GABA input. Next, we examine how neuron responds to both application of $[K^{+}{]}_{\mathrm{bath}}=3.0\;\;\mathrm{mM}$ and GABA input with ${\mathrm{HCO}}_{3}^{-}$ outflux.

### Competitive and synergistic interplay between GABA stimuli and potassium exchange regulates the neuron firing activity

**Figure 6.**
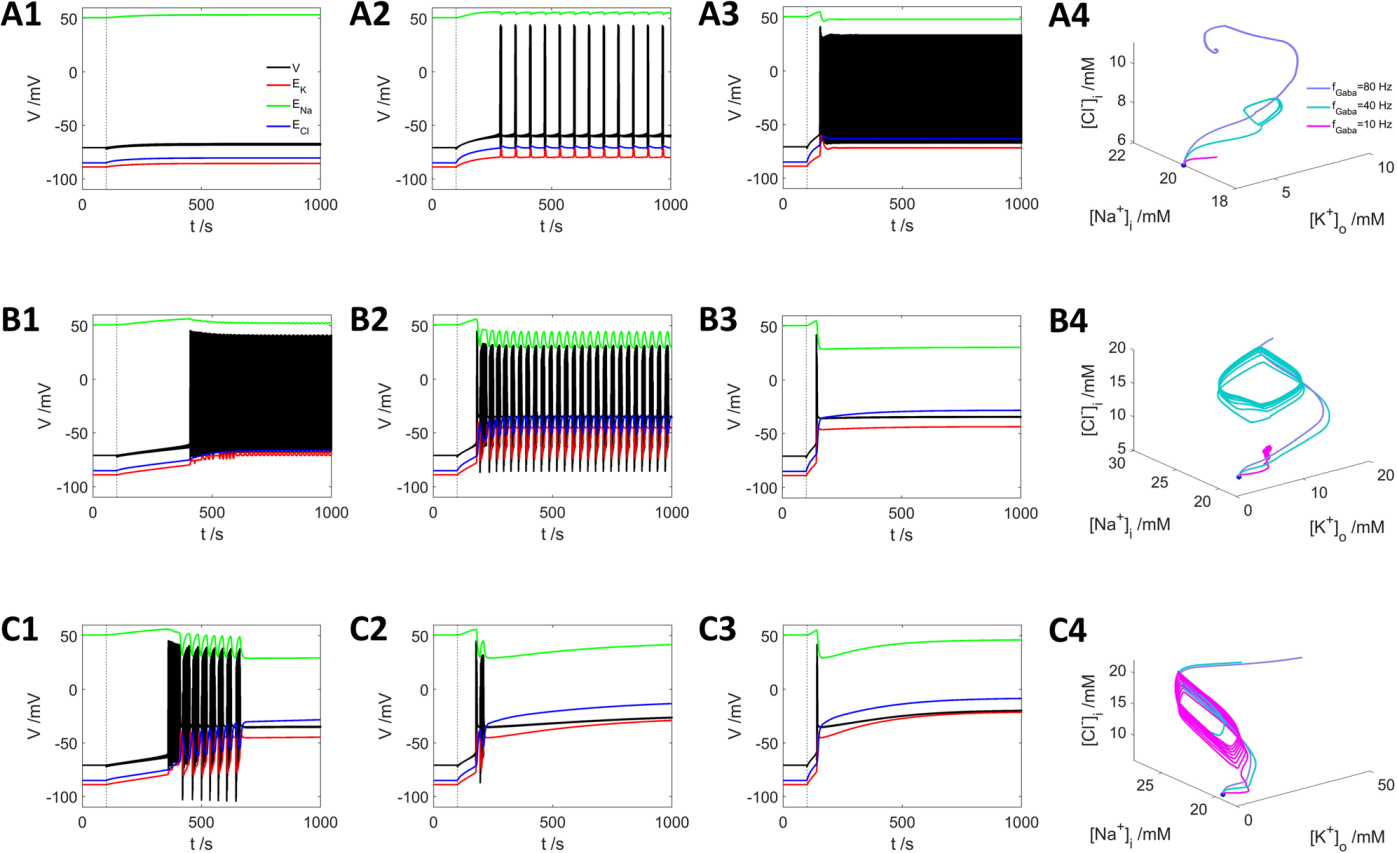
Neuron firing activities by applying different frequency of GABA spike train stimuli with different diffusion rate $\varepsilon_{k}$ to $[K^{+}{]}_{\mathrm{bath}}$. ***A1*–*3***, Neuron firing activities by stimuli of GABA input with 10, 40, and 80 Hz, respectively. ***A4***, The corresponding dynamic trajectories with 10 Hz (magenta), 40 Hz (cyan), and 80 Hz (light blue), with $\varepsilon_{k}=0.25\;s^{-1}$. ***B1*–*4***, Same as ***A1*–*4*** but with $\varepsilon_{k}=0.025\;s^{-1}$. ***C1*–*4***, Same as ***A1*–4** but with even lower $\varepsilon_{k}=0.0025\;\;s^{-1}$. ***A2***,**3**, ***B1*–*3***, ***C1*–*3*** share the same legend as ***A1***; and ***B4*** and ***C4*** share the same legend as ***A4***.

When the K^+^ exchange rate is high at $\varepsilon_{k}=0.25\;s^{-1}$ the neuronal response depends on the frequency of GABA spike stimulation (Fig. 6*A1–3*). In Figure 6*A1*, GABA spike input with 10 Hz did not cause the neuron to fire, indicating that the effect from $[K^{+}{]}_{\mathrm{bath}}$ to decrease $[Cl^{-}{]}_{i}$ is stronger than that of GABA stimuli to increase $[Cl^{-}{]}_{i}$. Therefore, the neuron goes to resting state with low $[Cl^{-}{]}_{i}$ (Fig. 6*A4*, magenta curve). Increasing GABA frequency to 40 Hz causes action potentials, showing that the accumulation of $[Cl^{-}{]}_{i}$ by GABA stimuli overcomes the effect of $[K^{+}{]}_{\mathrm{bath}}$ (Fig. 6*A4*, cyan curve with limit cycle). Further increasing the GABA frequency to 80 Hz causes more intensive firing activities as shown in Figure 6*A3*, demonstrating that the $[Cl^{-}{]}_{i}$ increase from GABA input is now much stronger, as can be seen from the dynamic trajectories in Figure 6*A4* with light blue curve.

With a lower K^+^ exchange rate $\left(\varepsilon_{k}=0.025\;s^{-1}\right)$, GABA stimulation with frequencies of 10, 40, and 80 Hz all trigger firing activities (Fig. 6*B1–3*), with earlier onset of firing and higher action potential frequencies. With 10 Hz stimulation, the neuron exhibits intensive firing activities (Fig. 6*B1*; Fig. 6*B4*, magenta curve). Increasing the frequency to 40 Hz causes periodic bursting interrupted with DB state (Fig. 6*B*2; Fig. 6*B4*, cyan curve); and with 80 Hz stimuli, the neuron immediately turns to the DB state after a few action potentials (Fig. 6*B*3; Fig. 6*B*4, light blue curve), indicating the overwhelming strength of GABA input over $[K^{+}{]}_{\mathrm{bath}}$.

Finally, with a very small exchange rate $\varepsilon_{k}=0.0025\;s^{-1}$, all GABA stimuli frequencies eventually lead to a DB state with different trajectories (Fig. 6*C1–4*).

**Figure 7.**
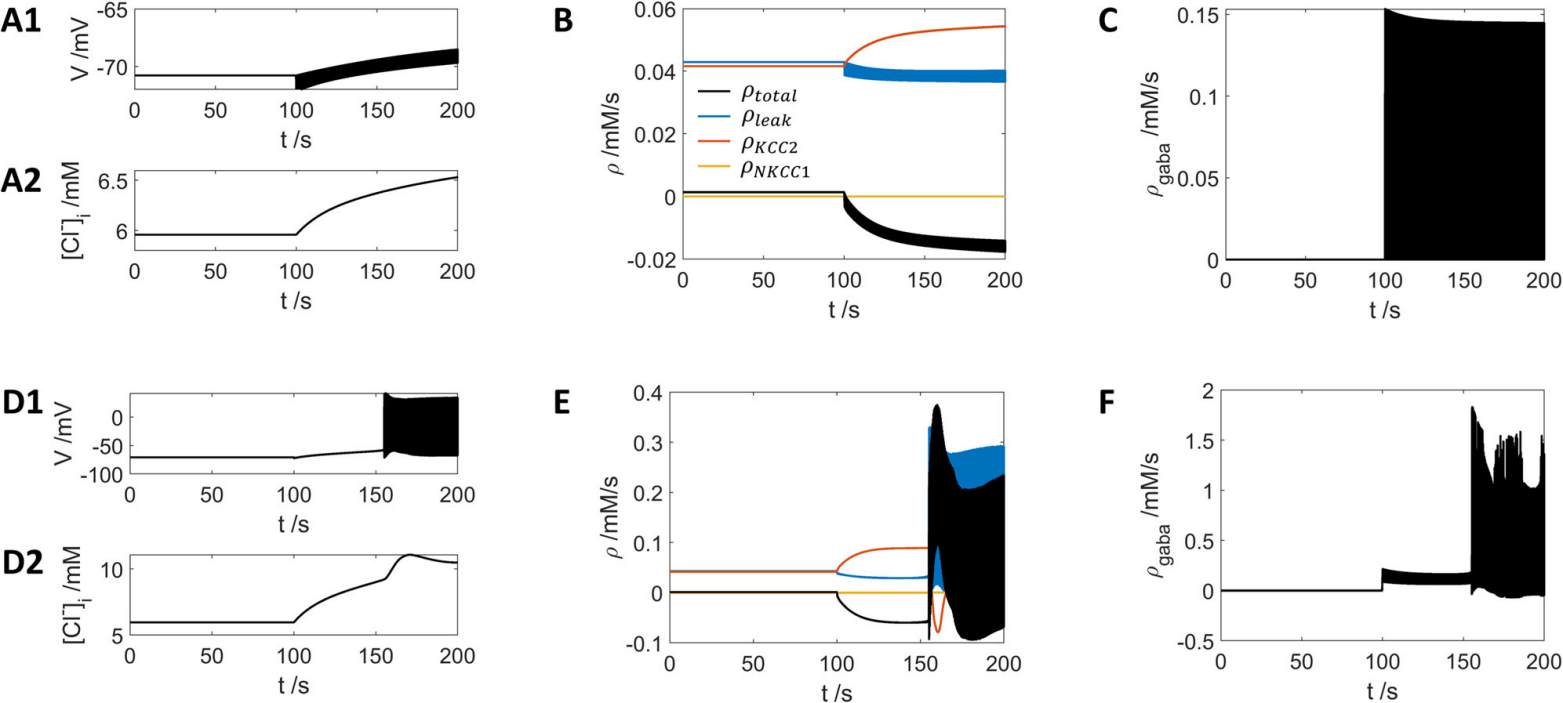
Competition between GABA input-induced increase of $[Cl^{-}{]}_{i}$ and exchange of $\;[K^{+}{]}_{o}$-induced decrease of $[Cl^{-}{]}_{i}$ from Figure 6*A1*,*3* for the first 200 s. ***A1***, The voltage dynamics; ***A2***, the dynamics of $[Cl^{-}{]}_{i}$; ***B***, the contribution to the decrease of $[Cl^{-}{]}_{i},$ where the red curve is contribution from KCC2; yellow curve is contribution from NKCC1, the blue curve is the contribution from chloride leak current, and the black curve is the summation of all the three contributions. ***C***, The contribution to the increase of $[Cl^{-}{]}_{i}$. ***D–F*** are similar to ***A–C*** but with 80 Hz stimuli as shown in Figure 6*A*3. Figure 7*E* shares the same legend as Figure 7*B*.

The mechanism affecting $[Cl^{-}{]}_{i}\;$ dynamics when GABA spike input is combined with $[K^{+}{]}_{o}\;$ exchange is shown in detail in Figure 7 by taking the example of Figure 6*A1* (Fig. 7*A–C*) and Figure 6*A3* (Fig. 7*D–F*). In Figure 7*B*, the contribution from KCC2 (red curve) increases, the contribution from NKCC1 (yellow curve) did not change, while the leak current (blue curve) decreases, all of which contribute to the $[K^{+}{]}_{o}$ exchange-induced decrease of $[Cl^{-}{]}_{i}$ (black curve); however, as shown in Figure 7*C*, the GABA input-induced $[Cl^{-}{]}_{i}\;$ current increases to a much larger degree than the black curve in Figure 7*B*; therefore, the GABA input-induced increase of $[Cl^{-}{]}_{i}\;$ wins over the $[K^{+}{]}_{o}$ exchange-induced decrease of $[Cl^{-}{]}_{i}$ and $[Cl^{-}{]}_{i}\;$ increases to a higher level (Fig. 7*A2*).

A similar phenomenon is shown in Figure 7*D–F* for the first 200 s from Figure 6*A3*. The GABA input-induced contribution of $[Cl^{-}{]}_{i}$ increase (Fig. 7*F*) is much larger than the $[K^{+}{]}_{\mathrm{bath}}$ application-induced decrease of $[Cl^{-}{]}_{i}$ (Fig. 7*E*, black curve); therefore, the $[Cl^{-}{]}_{i}\;$ increases as shown in Figure 7*D2*.

Our results show that the presence of SLE depends on the competition between two effects: one drives $[Cl^{-}{]}_{i}$ to lower levels because of the potassium exchange with $[K^{+}{]}_{\mathrm{bath}}$, and the counterbalancing one tends to elevate $[Cl^{-}{]}_{i}$ through GABA stimuli with ${\mathrm{HCO}}_{3}^{-}$ outflux. Smaller values of $\varepsilon_{K}$ decrease $[Cl^{-}{]}_{i}$ slowly, while larger values of $\varepsilon_{K}$ decrease $[Cl^{-}{]}_{i}$ fast, effectively preventing epileptic seizures at the single neuron level.

**Figure 8.**
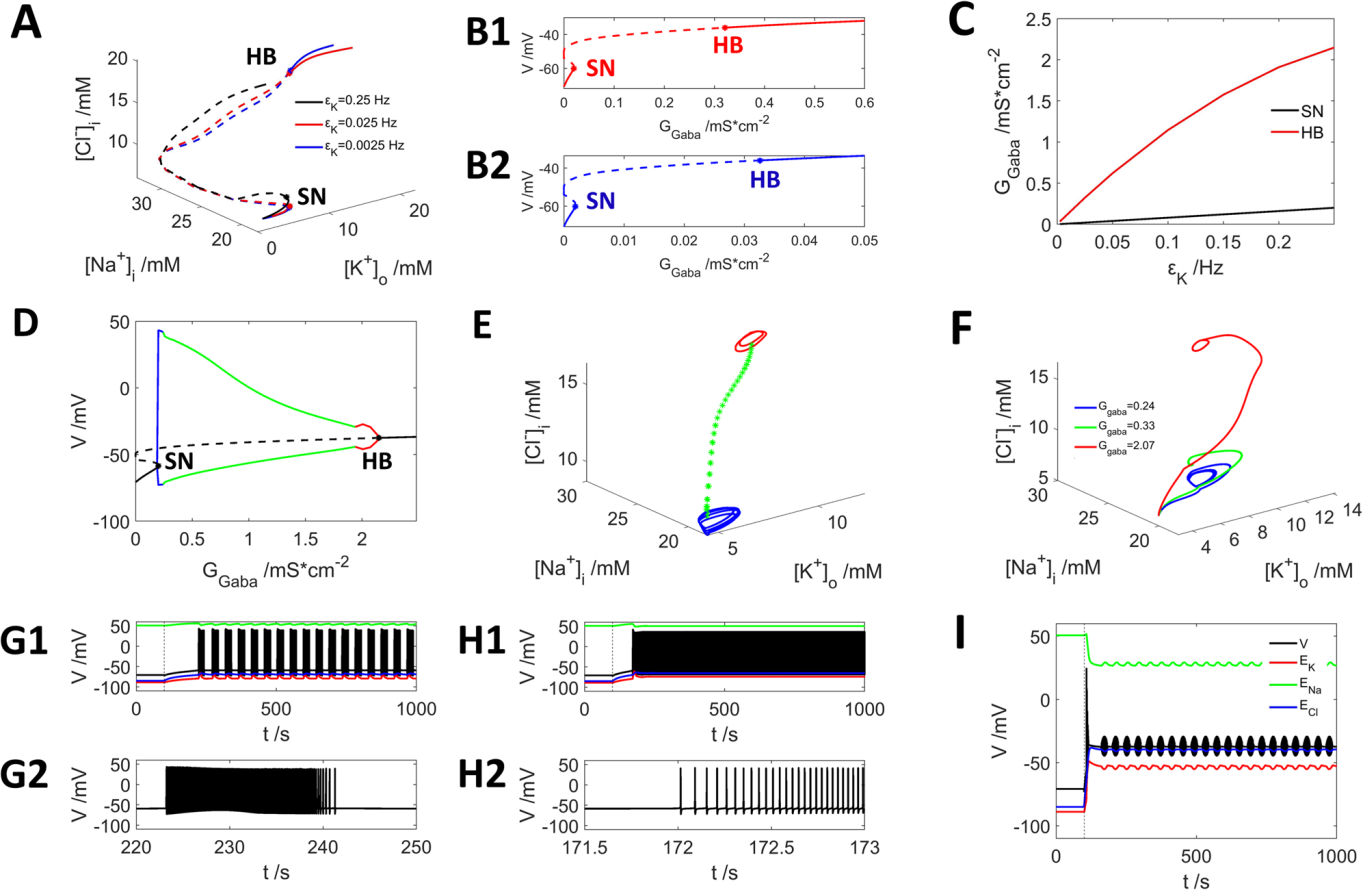
Neuron discharge state determined by competition between two parameters: step current input with constant $G_{\mathrm{GABA}}$ and potassium exchange rate $\varepsilon_{k}$. ***A***, Steady-state distribution of voltage with step current input of GABA and application of potassium diffusion with $[K^{+}{]}_{\mathrm{bath}}=3\;\mathrm{mM}$. Black, $\varepsilon_{k}=0.25\;s^{-1}$; red, $\varepsilon_{k}=0.025\;s^{-1}$; blue, $\varepsilon_{k}=0.0025\;s^{-1}$. ***B***, The projection of steady-state voltage onto the axis of strength of GABA step current, red, $\varepsilon_{k}=0.025\;s^{-1}$; blue, $\varepsilon_{k}=0.0025\;s^{-1}$. ***C***, The phase diagram for the SN and HB point with different $\varepsilon_{k}$ and $G_{\mathrm{GABA}}$. ***D***, Neuron firing patterns by different strength of GABA current input $\left(\varepsilon_{k}=0.25\;s^{-1}\right)$, blue, periodic firing; green, intensive firing; red, subthreshold periodic oscillation. ***E***, Dynamic trajectory in the phase space corresponding to the firing patterns shown in ***D*** (colored curves). ***F***, Examples of dynamic trajectories for neuron firing activity shown in ***G*** $\left(G_{\mathrm{GABA}}=0.24\;\mathrm{mS}\cdot cm^{-2}\right)$, ***H*** $\left(G_{\mathrm{GABA}}=0.33\;\mathrm{mS}\cdot cm^{-2}\right)$, ***I*** $\left(G_{\mathrm{GABA}}=2.07\;\mathrm{mS}\cdot cm^{-2}\right)$ corresponding to blue, green, and red curves, respectively. ***G1*** and ***H2*** show the firing activity at a smaller time scale. ***G1***, ***H1*** share the same legend as ***I***.

To quantitatively plot the parameter space within which the effect of GABA input wins out that from potassium exchange, or vice versa, we performed an analytical calculation of the steady-state distribution of the voltage with GABA step current input and potassium bath application with $[K^{+}{]}_{\mathrm{bath}}=3.0\;\mathrm{mM}$. Our results show that the system exhibits SN and HB bifurcations (Fig. 8*A*) and that the different potassium exchange rates give to different steady states (Fig. 8*A*, red, black, blue curves). For example, by comparing Figure 8*B1* $\left(\varepsilon_{k}=0.025\;s^{-1}\right)$ with Figure 8*B2* $\left(\varepsilon_{k}=0.0025\;s^{-1}\right)$, it is clear that with the higher $\varepsilon_{k}$ the neuron requires larger GABA stimuli to escape from the resting state and generate spiking than the condition with lower $\varepsilon_{k}$ value, and the same applies for HB point with the transition to DB state.

The dependence of the bifurcation points delineating the different firing regimes on $G_{\mathrm{GABA}}$ and exchange rate $\varepsilon_{k}$ is plotted in Figure 8*C*. The HB (red curve) point leading to DB state is much more sensitive to the exchange rate $\varepsilon_{k}$ than the SN (black curve).

In Figure 8*D–I*, we show examples of the neuron responses in the case of a large exchange rate of potassium with the bath $[K^{+}{]}_{\mathrm{bath}}=3\;\;\mathrm{mM}$, $\varepsilon_{k}\;=0.25\;s^{-1}$. The relationship between the membrane potential and $G_{\mathrm{GABA}}$ is plotted in Figure 8*D*, where the black curve indicates that the neuron requires at least $G_{\mathrm{GABA}}=0.20\;\mathrm{mS}\cdot cm^{-2}$ to generate action potentials, the blue curve (Fig. 8*D*,*E*) shows the periodic bursting activities (example in Fig. 8*G*; $G_{\mathrm{GABA}}=0.24\;\mathrm{mS}\cdot cm^{-2}$) and the green curve (Fig. 8*D*,*E*) the intensive firing of the neuron (example in Fig. 8*H*; $G_{\mathrm{GABA}}=0.33\;\mathrm{mS}\cdot cm^{-2}$). The envelop shrinks with increasing $G_{\mathrm{GABA}}$, and with very large $G_{\mathrm{GABA}}$ ∼1.8 mS cm^−2^, the neuron shows a subcellular periodic oscillation (example in Fig. 8*I*, periodic little bump; $G_{\mathrm{GABA}}=2.07\;\mathrm{mS}\cdot cm^{-2}$). Finally, the neuron enters the DB state with $G_{\mathrm{GABA}}=2.15\;\mathrm{mS}\cdot cm^{-2}$. The dynamic trajectories for Figure 8*G–I* are plotted in Figure 8*F* by blue, green, and red curves, respectively, showing that the stronger GABA input, the higher $[Cl^{-}{]}_{i}$ becomes, leading to a more depolarized state.

### Rebound behavior after termination of GABA input

**Figure 9.**
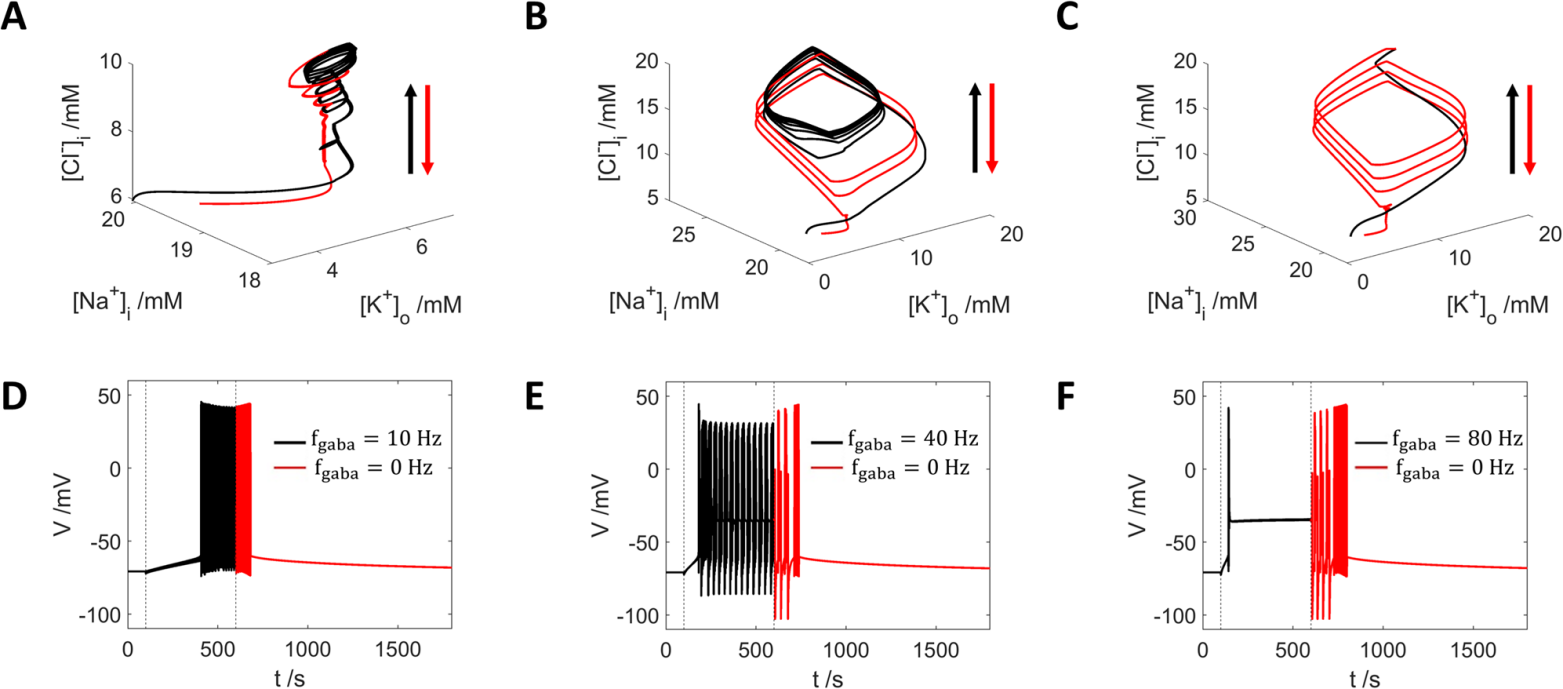
Rebound firing activity upon termination of GABA stimuli. $[K^{+}{]}_{\mathrm{bath}}\;=3.0\;\;\mathrm{mM},\;\;\varepsilon_{k}=0.025\;s^{-1}$. ***A–C***, Dynamic trajectory of neuron firing activity by GABA stimuli during 100–600 s with 10, 40, and 80 Hz, respectively. Black arrow, the direction of the dynamic trace during GABA stimulation; red arrow, dynamic trace after ending the GABA stimuli. ***D–F***, Neuron firing activity with dynamics of voltage by GABA stimuli of 10, 40, and 80 Hz, respectively. The first dashed vertical line shows the onset of GABA input at *t* = 100 s; the second vertical dashed line indicates the end of stimulation at *t* = 600 s. Black curve, neuronal firing activities during GABA stimulation; red curve, neuronal rebound behavior after end of stimulation. ***A*** and ***D***, ***B*** and ***E***, and ***C*** and ***F*** share the same legends, respectively.

We complete the analysis of the neuronal response to GABA input by demonstrating the rebound behavior after the termination of a GABA spike train stimuli, as reported experimentally (Perkel and Mulloney, 1974; Kuwada and Batra, 1999; Felix et al., 2011; Adhikari et al., 2012; Chang et al., 2018). We set $[K^{+}{]}_{\mathrm{bath}}=3.0\;\mathrm{mM}$ during the whole simulation process and apply a spike train of GABA stimuli during 100–600 s (Fig. 9). As can be seen in Figure 9*D*, with 10 Hz stimulation the neuron slowly depolarizes and then starts firing intensively (black curve), and the dynamic trajectory is evolving upward with increasing $[Cl^{-}{]}_{i}$ showing a limit cycle in the phase space (Fig. 9*A*, black curve). When the GABA stimulation terminates at 600 s, the neuron shows rebound activity (Fig. 9*A*,*D*, red curve) and the trajectory evolves downward to lower $[Cl^{-}{]}_{i}$ and ends up with the resting state.

With higher frequency of GABA stimuli (40 Hz), the neuron exhibits intensive firing and then periodic bursting with DB activities (Fig. 9*B*,*E*, black curves). Similar to the 10 Hz case, the neuron generates rebound firing activities with larger limit cycles after withdrawn GABA spike input; and it evolves toward the lower $[Cl^{-}{]}_{i}\;$ direction and a resting state (Fig. 9*B*,*E*, red curves). At 80 Hz stimulation, the neuron immediately exhibits the firing activity followed by a stable DB state (Fig. 9*C*,*F*, black curves), the rebound activity (Fig. 9*C*,*F*, red curves) shows intensive firing but leads to lower $[Cl^{-}{]}_{i}$ and eventually neuron goes to resting state. Our results demonstrate the basic mechanism of opposing effects on $[Cl^{-}{]}_{i}$: by withdrawing GABA input, the drive to increase $[Cl^{-}{]}_{i}$ suddenly disappears, and the potassium exchange-induced decrease of $[Cl^{-}{]}_{i}$ dominates; thus, the neuron evolves into the resting state.

## Discussion

In summary, we describe how the influence of increasing $[Cl^{-}{]}_{i}$ on intrinsic neuronal properties can be characterized by SN and HB bifurcations, similar to the voltage-dependent dynamics (Rinzel and Ermentrout, 1998). Our results reveal that neuron firing activity is regulated by the interplay between two opposing effects: upregulation of $[Cl^{-}{]}_{i}$ by GABA input with ${\mathrm{HCO}}_{3}^{-}$ efflux and downregulation of $[Cl^{-}{]}_{i}\;$by potassium exchange to a low $[K^{+}{]}_{\mathrm{bath}}$. Our results elucidate the crucial role of $[Cl^{-}{]}_{i}$ in enhancing epileptic SLEs within the framework of a single-compartment neuron model.

Epileptic seizures are intricate pathological phenomena influenced by many factors (Timofeev and Steriade, 2004; Moshé et al., 2015; Gentiletti et al., 2022; Wenzel et al., 2023). Our single-neuron model demonstrates that GABA input with ${\mathrm{HCO}}_{3}^{-}$ efflux can increase the intracellular chloride concentration and lead to a spectrum of distinct firing activities, comparable with SLE, and consistent with experimental observations of elevated intracellular chloride concentrations by external stimuli (Timofeev et al., 2002; Somjen, 2004; Lillis et al., 2012; Staley, 2015; Magloire et al., 2019). However, the conclusions derived from various experiments regarding the role of inhibitory neurons in epilepsy showed inconsistent results (de Curtis and Avoli, 2016; Weiss, 2023; Wenzel et al., 2023). It is well known that inhibitory neurons have the capacity to mitigate epileptic seizures by hyperpolarizing excitatory neurons and numerous experimental and modeling studies have underscored that disruption of this delicate excitatory–inhibitory balance can precipitate epileptic seizures (McCormick and Contreras, 2001; Liu et al., 2020; van Hugte et al., 2023). Additionally, prolonged GABA input can result in the accumulation of $[Cl^{-}{]}_{i}$, thereby facilitating epileptic seizures (Trevelyan et al., 2006, 2007; Ben-Ari et al., 2007; Burman et al., 2019). Our findings can explain this double role of GABA input. Upon GABA receptor activation, if there is only chloride influx, then the effect of GABA will be inhibition provided that the reversal potential of chloride remains below the membrane potential. If there is also ${\mathrm{HCO}}_{3}^{-}$ efflux upon GABA receptor activation, then it will turn the inhibition of GABA into excitation by depolarization, caused by the elevated reversal potential of ${\mathrm{HCO}}_{3}^{-}$. The permeability of ${\mathrm{HCO}}_{3}^{-}$ is between 0.18 and 0.44 of the chloride permeability (Bormann et al., 1987; Kaila and Voipio, 1987; Fatima-Shad and Barry, 1993; Kaila et al., 1997, 2014), and we assumed a constant ${\mathrm{HCO}}_{3}^{-}$ reversal potential $E_{\mathrm{HC}O_{3}}$ of −13 mV. This value may change during neural activity as the dynamics of $E_{\mathrm{HC}O_{3}}$ depend on the detailed chemical reaction and transport of ${\mathrm{HCO}}_{3}^{-}$ in neuronal systems. It is possible that the concentration of ${\mathrm{HCO}}_{3}^{-}$ varies, allowing for dynamic modulation of the depolarizing effect of ${\mathrm{HCO}}_{3}^{-}$ (Farrant and Kaila, 2007). Possible mechanisms might include secondary active ${\mathrm{HCO}}_{3}^{-}$ uptake via electroneutral and electrogenic $Na^{+\;}{/\mathrm{HCO}}_{3}^{-}$ symporters (Hübner and Holthoff, 2013) and intracellular pH changes by the carbonic anhydrases (Sinning and Hüebner, 2013). Elucidating ${\mathrm{HCO}}_{3}^{-}$ dynamics in the brain is an important question for future investigations.

In addition, the potassium homeostatic mechanism serves as a regulator of $[Cl^{-}{]}_{i}$ during spontaneous discharge behaviors. In our work, we have demonstrated that it is the interplay between potassium homeostasis and GABA stimuli with ${\mathrm{HCO}}_{3}^{-}$ efflux that shapes neuron firing activities. Nevertheless, the precise dynamical properties of the potassium homeostatic mechanism remain elusive, with potentially important role of astrocytes (Bradbury and Davson, 1965; Volman et al., 2012; Chizhov et al., 2019; Palabas et al., 2022). Our study demonstrates that a larger potassium exchange rate (denoted as $\varepsilon_{K}$) can mitigate epileptic discharges by shortening the maximum seizure duration and drive the neuron firing activities to a state with lower $[Cl^{-}{]}_{i}$. The strength and timing of the potassium homeostasis will be critically important for the preventive treatment of epilepsy.

Existing reports indicate that potassium concentration $[K^{+}{]}_{o}$ is maintained at ∼3 mM in vivo (Raimondo et al., 2015; Staley, 2015), while the corresponding value of $\varepsilon_{K}$ remains largely unknown. The physiological significance of the diffusion coefficient $\varepsilon_{K}$ covers neuron–glia interactions and neuron–capillary interactions. Consequently, the means to expediently reduce extracellular potassium concentrations, whether through blood vessels or alternative mechanisms (Somjen, 2004; de Curtis et al., 2018), seem critically important. Our work underscores the importance of precise measurements of the strength and speed of the potassium homeostatic mechanism in physiological environments to better understand seizure events.

We examine the role of the K-Cl cotransporter NKCC1 and KCC2 in regulating SLE and $[Cl^{-}{]}_{i}$ dynamics in Figures 5 and 7. KCC2 transport one chloride ion and one potassium ion into the extracellular space, making it a potentially effective means of reducing $[Cl^{-}{]}_{i}$ (Payne et al., 2003; Gamba, 2005; Kahle et al., 2008; Blaesse et al., 2009; Kaila et al., 2014), especially with exchange process with $[K^{+}{]}_{\mathrm{bath}}$. Some studies have indicated at the possibility of pathological alterations in KCC2 contributing to certain behaviors in neural networks (Kaila et al., 2014; Moore et al., 2017; González et al., 2018). We found that the relative strength of NKCC1 and KCC2 contributes to the change of chloride concentration and that the $Cl^{-}$ leak current is also quite important. How the cotransporters of NKCC1 and KCC2 coherently can regulate the dynamics of chloride and mitigate epileptic seizures, and how their roles change in various physiological situations, still need further investigation.

During epileptic seizures, changes in ion concentrations lead to alterations in intracellular and extracellular osmotic pressures, resulting in dynamic fluctuations in the ratio of neuronal intracellular to extracellular volume (McBain et al., 1990; Østby et al., 2009; Hrabetova et al., 2018). The volume factor in the model indicates how strongly intracellular ion concentration variations affect the reversal potential. In physiologically realistic systems, the volume factor changes with neuronal firing properties, which involves an additional level of complexity that is challenging to simulate and analyze accurately. In our study we assumed the volume factor is fixed at β=7. We also tested our results with different volume factors, the fixed-point solution of the neuron system and the SN and HB points still exist but the distribution of them at each level of chloride concentration varies with β. The neuron exhibits qualitatively similar results yet distinct discharge patterns based on the SN and HB points in the system (data not shown).

## Synthesis

Reviewing Editor: Masha Prager-Khoutorsky, McGill University Decisions are customarily a result of the Reviewing Editor and the peer reviewers coming together and discussing their recommendations until a consensus is reached. When revisions are invited, a fact-based synthesis statement explaining their decision and outlining what is needed to prepare a revision will be listed below. The following reviewer(s) agreed to reveal their identity: NONE. Note: If this manuscript was transferred from JNeurosci and a decision was made to accept the manuscript without peer review, a brief statement to this effect will instead be what is listed below.

Synthesis of Reviews:

The reviewers appreciate the amount of work the authors put into the rebuttal for J Neurosci. While the reviewers are not entirely satisfied with the justification of the authors for not using excitation in the model since excitation is critical for epilepsy, they believe that this is a very interesting paper that will be important for researchers in the field.
